# Serum globulin is a novel predictor of mortality in patients undergoing peritoneal dialysis

**DOI:** 10.1038/s41598-023-27688-z

**Published:** 2023-01-20

**Authors:** Yao-Peng Hsieh, Shr-Mei Tsai, Chew-Teng Kor, Ping-Fang Chiu

**Affiliations:** 1 Division of Nephrology, Department of Internal Medicine, Changhua Christian Hospital, 135 Nanxiao Street, Changhua City, 500 Taiwan; 2 School of Medicine, Kaohsiung Medical University, Kaohsiung, Taiwan; 3 School of Medicine, Chung Shan Medical University, Taichung, Taiwan; 4 Department of Post Baccalaureate Medicine, College of Medicine, National Chung Hsing University, Taichung, Taiwan; 5 Department of Nursing, Changhua Christian Hospital, Changhua, Taiwan; 6 Department of Recreation and Holistic Wellness, MingDao University, Changhua, Taiwan

**Keywords:** Nephrology, Kidney, Kidney diseases, Renal replacement therapy

## Abstract

Serum globulin, which is composed mainly of immunoglobulins and acute phase proteins, can be considered as reflecting the inflammatory state. We conducted the present study to investigate the role of globulin in mortality risk in patients undergoing peritoneal dialysis (PD). The study participants were categorized by the median globulin value (2.8 g/dL) as the high globulin group (≥ 2.8 g/dL), and low globulin group (< 2.8 g/dL). Serum globulin is calculated by the equation: (serum total protein-serum albumin). The area under the curve (AUC) by the receiver operating characteristics curve analysis was calculated to compare the mortality prediction capacity of globulin with that of ferritin, and WBC counts. Among the 554 patients, 265 (47.83%) were men, the mean age was 52.91 ± 15.54 years and the body mass index was 23.44 ± 3.88 kg/m^2^. Multivariate Cox models showed the high globulin group had higher mortality risks of all-cause and cardiovascular disease (CVD), compared with the low globulin group with adjusted HRs of 2.06 (95% CI 1.39–3.05) and 1.94 (95% CI 1.18–3.16), respectively. The AUC of univariate and multivariate models for all-cause mortality resulted in higher AUC values for globulin than for ferritin and white blood cell (WBC) counts. In patients undergoing PD, the serum globulin can serve as a novel and independent determinant of predicting overall and CVD- associated mortality.

## Introduction

Approximately 11.5% of the general population had chronic kidney disease (CKD), which also occurred in 40% of diabetic patients in the United States^1,2^. Peritoneal dialysis (PD) is one of the accepted renal replacement therapies for patients with end-stage renal disease (ESRD) worldwide. The mortality rate of those patients remains much higher than the general population despite the modern improvement in medical science. CKD is an equivalent of cardiovascular disease, which was the leading cause of mortality among patients with CKD and accounted for about 40% of patient deaths^3,4^. Persistent low-grade inflammation, observed in the clinical setting of CKD, particularly ESRD, plays a pivotal role in the pathogenesis of high cardiovascular morbidity and mortality in this population^5,6^.

Inflammation had been reportedly modifying and catalyzing the vicious cycle of risk factors in ESRD, including atherosclerosis, vascular calcification and protein-energy wasting (PEW), leading to the tremendous adverse outcomes^6^. Therefore, inflammation has emerged as a therapeutic target through various nutritional and pharmacological interventions with the goal of improving the frustrating prognosis. C-reactive protein (CRP), a member of the pentraxin protein family produced by liver cells, is the most studied inflammatory marker because of its wide availability and low cost^7^. A post hoc analysis of a randomized controlled trial involving 4038 diabetic patients with anemia and CKD reported that baseline CRP levels are associated with the future development of ESRD and the composite of death or ESRD^8^.

Aside from CRP, several biochemical inflammatory markers, such as interleukin-6, interleukin-18, endoxin and gelsoin, had been shown to predict cardiovascular risk and mortality in patients with CKD^5,9–11^. Serum globulin consisting primarily of immunoglobulins and acute phase proteins can be considered to reflect an inflammatory state. However, there is a lack of research on the relationship between serum globulin and mortality in patients undergoing PD. Therefore, we conducted the present study to test the hypothesis that higher serum globulin levels predicted higher risk of death.

## Results

### Patients’ baseline characteristics

A total of 554 patients on PD were left for the final analysis with 257 patients in the low globulin group (< 2.8 g/dL) and 297 in the high globulin group (≧2.8 g/dL). The mean follow-up time was 3.87 ± 3.15 years. Among the study participants, 265 (47.83%) were men, the mean age was 52.91 ± 15.54 years and the BMI was 23.44 ± 3.88 kg/m^2^ (Table 1.). The vast majority of them never smoked (n = 456, 82.31%) and had primary school (n = 176, 31.77%) as the educational level. Chronic glomerulonephritis, diabetes mellitus and hypertension were the three major causes of CKD. The low globulin group had higher proportions of using ACE inhibitors/ARB, ESA, and calcium channel blocker, and having hypertension compared with the high globulin group. In terms of laboratory data, serum creatinine and phosphorus levels were higher in the low globulin group, and ALP, triglyceride, hemoglobin, and WBC count levels were lower than in the high globulin group. In addition, the low globulin group also had higher daily nPNA.

**Table 1:** Patients’ demographic and clinical data by the median globulin value at study entry.

	Globulin < 2.8 (g/dL)	Globulin ≥ 2.8 (g/dL)	*P* value
Sample size	257	297	–
Demographic			
Gender (% male)	135 (52.53%)	130 (43.77%)	0.040
Age (years)	49.78 ± 14.79	55.62 ± 15.69	< 0.001
Body mass index (kg/m^2^)	23.53 ± 3.88	23.36 ± 3.89	0.607
Smoker			
Never	209 (81.32%)	247 (83.16%)	0.649
Current	8 (3.11%)	4 (1.35%)	0.258
Ever	40 (15.56%)	46 (15.49%)	1.000
Educational level			
Illiteracy	18 (7%)	48 (16.16%)	0.001
Primary school	79 (30.74%)	97 (32.66%)	0.695
Junior high school	36 (14.01%)	48 (16.16%)	0.558
Senior high school	64 (24.9%)	62 (20.88%)	0.305
College and above	60 (23.35%)	42 (14.14%)	0.007
Status ahead of PD			
Pre-dialysis	216 (84.05%)	246 (82.83%)	0.787
Hemodialysis	41 (15.95%)	51 (17.17%)	0.787
The causes of CKD			
Chronic glomerulonephritis	96 (37.35%)	81 (27.27%)	0.014
Hypertension	46 (17.9%)	51 (17.17%)	0.910
Diabetes mellitus	62 (24.12%)	93 (31.31%)	0.074
Others	53 (20.62%)	72 (24.24%)	0.36
Medications			
ACE inhibitor/ARB	174 (67.7%)	169 (56.9%)	0.009
Diuretics	121 (47.08%)	145 (48.82%)	0.683
Erythropoiesis-stimulating agents	246 (95.72%)	271 (91.25%)	0.035
Vitamin D	67 (26.07%)	87 (29.29%)	0.398
Calcium channel blocker	194 (75.49%)	195 (65.66%)	0.012
Comorbid conditions			
Hypertension	228 (88.72%)	243 (81.82%)	0.023
Diabetes mellitus	76 (29.57%)	110 (37.04%)	0.064
Cardiovascular disease	84 (32.68%)	116 (39.06%)	0.119
Hyperlipidemia	65 (25.29%)	77 (25.93%)	0.865
Cancer	12 (4.7%)	12 (4.0%)	0.717
Autoimmune disease	16 (6.2%)	18 (6.1%)	0.936
Laboratory data			
Globulin (g/dL)	2.5 (2.3, 2.6)	3.1 (2.9, 3.5)	< 0.001
Serum albumin (g/dL)	3.3 (2.8, 3.7)	3.3 (2.9, 3.8)	0.234
ALP(U/L)	90 (64.2, 128)	100 (71, 160)	0.002
Calcium (mg/dL)	8.3 (7.9, 8.8)	8.4 (7.9, 8.9)	0.285
Cholesterol (mg/dL)	181 (154, 218)	186 (154, 219)	0.779
Creatinine (mg/dL)	10.1 (8.43, 12.4)	9.14 (7.67, 11)	< 0.001
Ferritin (ng/mL)	252.2 (130.2, 424.39)	265.7 (132.2, 552.2)	0.086
GPT (U/L)	16 (12, 23)	16 (12, 23)	0.571
Hemoglobin (g/dL)	8.6 (7.6, 9.4)	8.8 (8, 9.5)	0.042
Intact PTH (pg/mL)	343.5 (209.4, 557.5)	316 (182, 501)	0.178
Phosphorus (mg/dL)	5.6 (4.8, 6.4)	5.3 (4.5, 6.1)	0.006
Triglyceride (mg/dL)	114 (83, 155)	124 (95, 173)	0.013
WBC count (× 10^3/μL)	6.9 (5.5, 7.9)	7.49 (6.2, 9.2)	< 0.001
PD-related parameters			
D/P creatinine at 4 h	0.68 (0.6, 0.76)	0.67 (0.6, 0.76)	0.402
24 h urine volume (L)	0.9 (0.5, 1.3)	0.86 (0.5, 1.25)	0.361
Weekly total Kt/V urea	2.04 (1.7, 2.35)	2.02 (1.74, 2.28)	0.658
Daily nPNA (g/kg)	1.07 (0.85, 1.26)	1.02 (0.84, 1.18)	0.030
Residual renal function (mL/min/1.73 m^2^)	2.89 (2, 3.94)	2.98 (2.11, 3.84)	0.744

Values are expressed as mean ± SD, median (interquartile range) or number (percentage).

*ACE inhibitor* angiotensin-converting enzyme inhibitor, *ARB* angiotensin II receptor blocker, *BMI* body mass index, *GPT* glutamic-pyruvic transaminase, *WBC* white blood cell count, *PTH* parathyroid hormone, *ALP* alkaline phosphate, *nPNA* normalized protein nitrogen appearance, *D/P creatinine* dialysate-to-plasma creatinine ratio.

### Association of globulin with all-cause mortality

**Figure 1:**
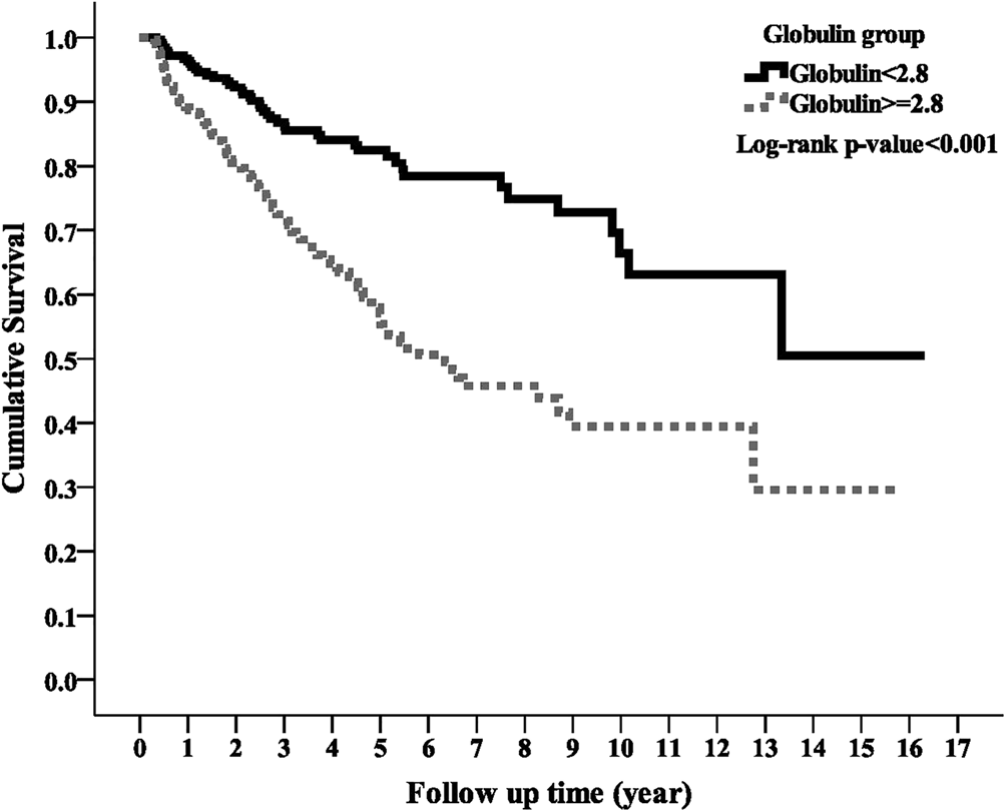
Kaplan–Meier curve of overall patient survival according to the globulin groups (log-rank test, *p* < 0.001).

During the study period, the high globulin group had a significantly higher all-cause mortality rate than the low globulin group (n = 106, 35.69% vs. n = 45, 17.51%; *p* < 0.001). Kaplan–Meier survival curve showed the high globulin group had lower survival rate than the low globulin group (Fig. 1; log-rank test, *p* < 0.001). Both univariate and multivariate Cox models showed the high globulin group had higher all-cause mortality risk compared with the low globulin group with an adjusted HR of 2.06 (95% CI 1.39–3.05) in the fully adjusted model (Table 2). The hazard ratios for all variables included in the model 5 were provided as supplementary materials (Supplementary Table 1). The sensitivity tests yielded similar results, which corroborated the primary results showing globulin was an independent covariate for all-cause mortality.

**Table 2:** Univariate and multivariate Cox regression models of mortality for the globulin groups.

	All-cause mortality		CVD mortality	
	Hazard ratio (95% CI)	*p* value	Hazard ratio (95% CI)	*p* value
(A) Median globulin (Globulin ≥ 2.8 vs. Globulin < 2.8)				
Univariate model	2.57 (1.81, 3.65)	< 0.001	2.34 (1.51, 3.60)	< 0.001
Model 1	1.94 (1.36, 2.77)	< 0.001	1.81 (1.16, 2.81)	0.008
Model 2	1.91 (1.33, 2.74)	< 0.001	1.76 (1.12, 2.77)	0.014
Model 3	1.99 (1.38, 2.88)	< 0.001	1.91 (1.21, 3.03)	0.006
Model 4	2.10 (1.45, 3.05)	< 0.001	1.98 (1.24, 3.15)	0.004
Model 5	2.06 (1.39, 3.05)	< 0.001	1.94 (1.18, 3.16)	0.009
(B) sensitivity tests				
(i) Globulin as a continuous variable	2.19 (1.59, 3.02)	< 0.001	2.71 (1.75, 4.19)	< 0.001
(ii) Globulin tertiles				
First tertile (Globulin < 2.6)	1		1	
Second tertile (2.6 ≤ Globulin < 3.0)	2.32 (1.35, 3.99)	0.002	2.07 (1.05, 4.08)	0.035
Third tertile (Globulin ≥ 3.0)	2.97 (1.82, 4.84)	< 0.001	2.71 (1.46, 5.03)	0.002
P for trend	–	< 0.001	–	0.007
(iii) optimal globulin by ROC analysis	2.06 (1.39, 3.05)	< 0.001	1.94 (1.18, 3.16)	0.009
(C) Exclude patients who have cancer or autoimmune disease				
Median globulin	1.90 (1.27, 2.86)	0.002	1.84 (1.10, 3.07)	0.020
Globulin as a continuous variable	1.93 (1.36, 2.76)	< 0.001	2.17 (1.36, 3.46)	0.001

Model 1: globulin, age, sex, BMI, smoking status, the cause of chronic kidney disease, the status ahead of peritoneal dialysis and educational level.

Model 2: Model 1 plus medications (ACE inhibitors/ARB, Diuretic, vitamin D, erythropoiesis-stimulating agents, and calcium channel blockers).

Model 3: Model 2 plus comorbidities (diabetes mellitus, hypertension, hyperlipidemia, cancer, autoimmune disease and cardiovascular disease).

Model 4: Model 3 plus PD related parameters (weekly total Kt/V urea, nPNA, D/P creatinine at 4 h, 24 h urine output, and residual renal function).

Model 5: model 4 plus laboratory data (albumin, creatinine, alkaline-phosphate, GPT, WBC counts, hemoglobin, ferritin, cholesterol, triglyceride, intact PTH, calcium and phosphorus).

The optimal cut-off value of globulin by ROC curve analysis is the same as the median globulin (2.8 mg/dL).

### Association of globulin with cardiovascular disease mortality

**Figure 2:**
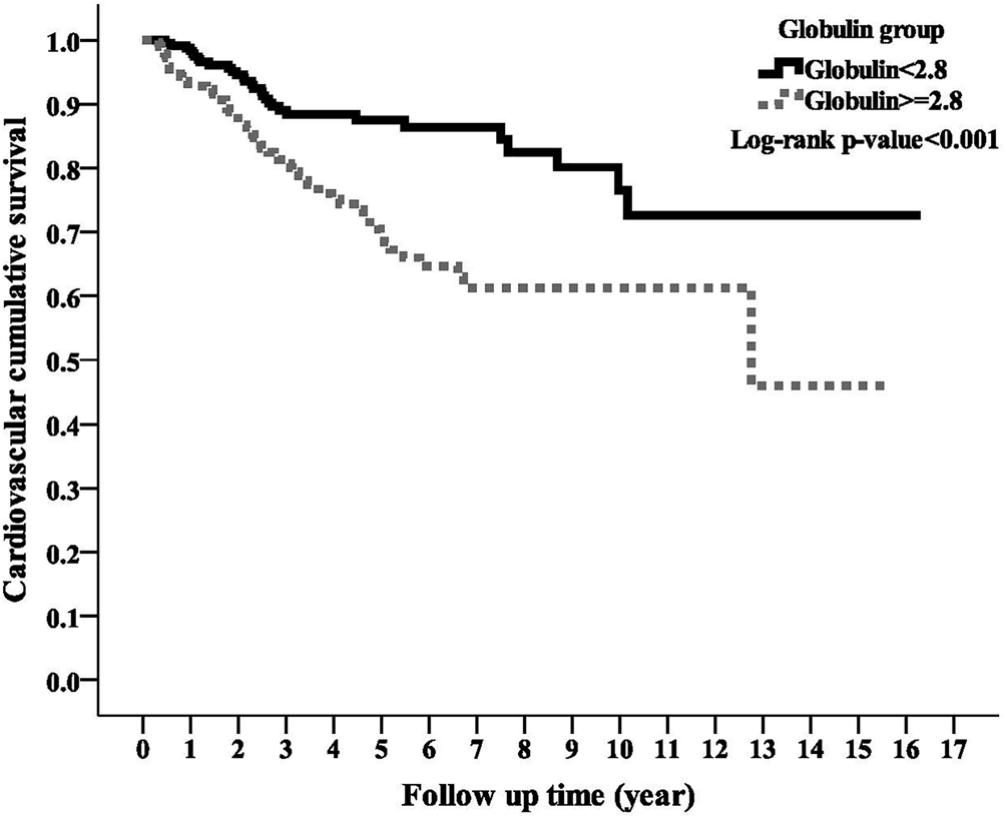
Kaplan–Meier curve of cumulative cardiovascular disease survival according to the globulin groups (log-rank test, *p* < 0.001).

During the study period, the high globulin group had a significantly higher cardiovascular disease mortality rate than the low globulin group (n = 65, 21.89% vs. n = 30, 11.67%; *p* < 0.001). Kaplan–Meier survival curve showed the high globulin group had lower cardiovascular survival rate than the low globulin group (Fig. 2; log-rank test, *p* < 0.001). Both univariate and multivariate Cox models showed the high globulin group had higher CVD mortality risk compared with the low globulin group with an adjusted HR of 1.94 (95% CI 1.18–3.16) in the fully adjusted model (Table 2). Consistent results were produced by the sensitivity tests, which corroborated the primary results showing globulin was an independent covariate for CVD mortality.

**Figure 3:**
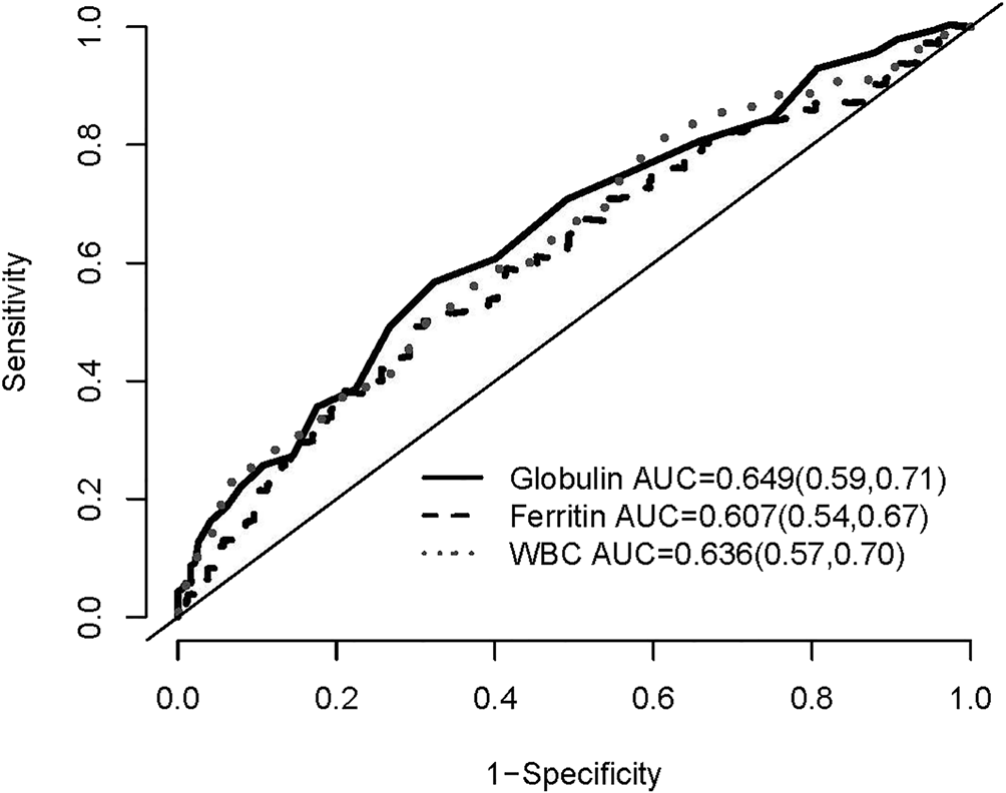
The area under the curve (AUC) by the receiver operating characteristics (ROC) curve analysis of the 3-year all-cause mortality for globulin, ferritin, and WBC counts. Numbers in parentheses indicate 95% confidence intervals.

**Figure 4:**
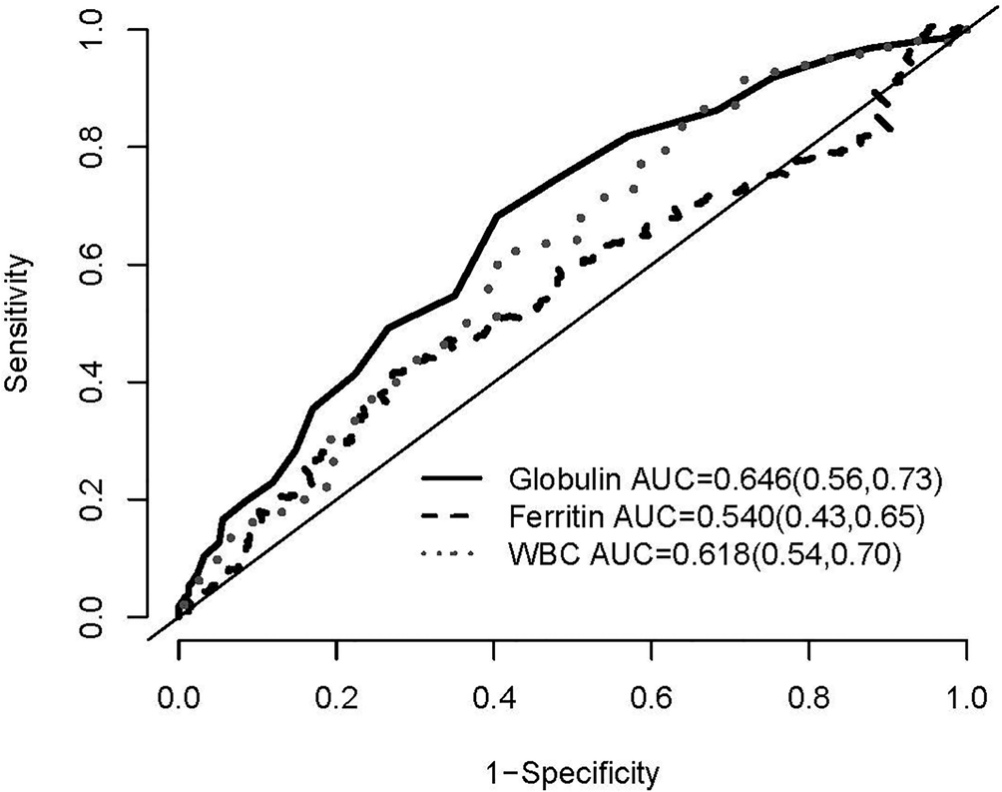
The area under the curve (AUC) by the Receiver Operating Characteristics (ROC) curve analysis of the overall all-cause mortality for globulin, ferritin, and WBC counts. Numbers in parentheses indicate 95% confidence intervals.

**Figure 5:**
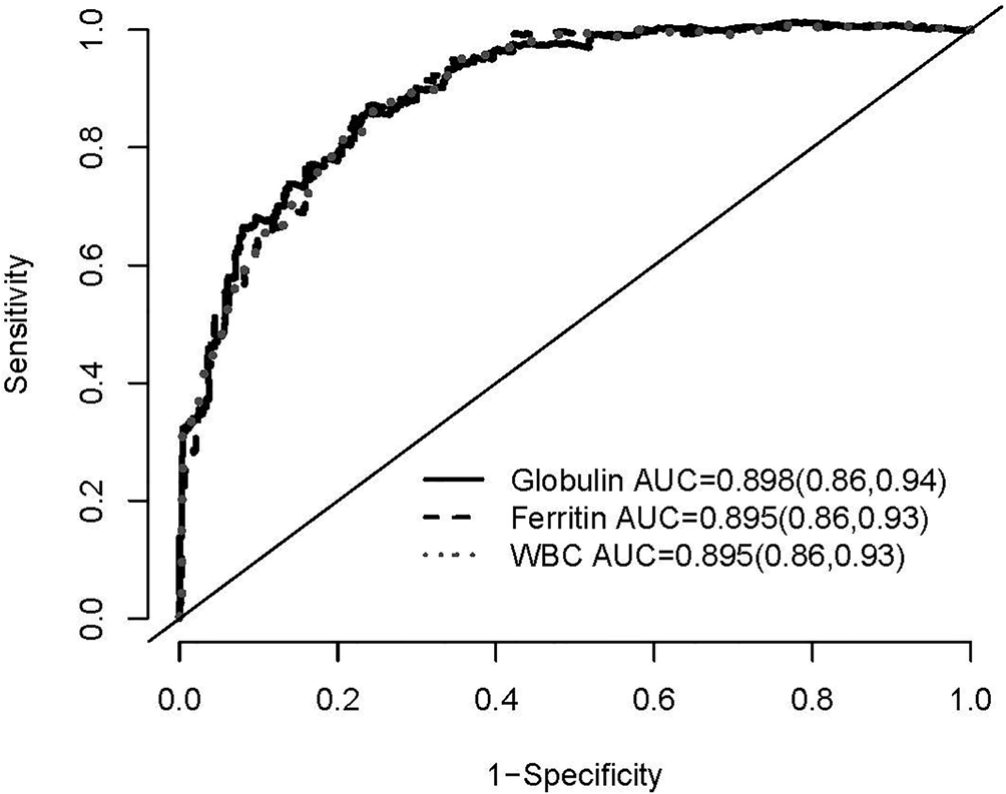
The area under the curve (AUC) by the Receiver Operating Characteristics (ROC) curve analysis of the 3-year all-cause mortality for variables in model 4 plus globulin, ferritin, and WBC counts, respectively. Numbers in parentheses indicate 95% confidence intervals.

**Figure 6:**
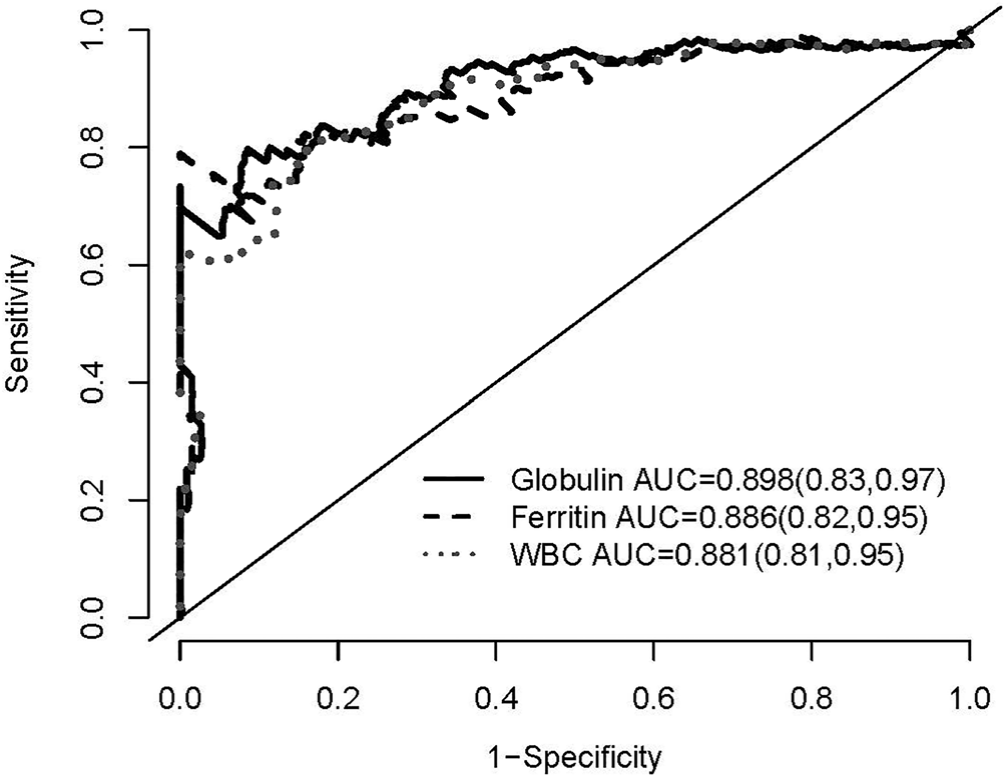
The area under the curve (AUC) by the Receiver Operating Characteristics (ROC) curve analysis of the overall all-cause mortality for variables in model 4 plus globulin, ferritin, and WBC counts, respectively. Numbers in parentheses indicate 95% confidence intervals.

### Association of globulin with mortality after excluding patients with malignancy or autoimmune disease

After excluding those patients who had malignancy or autoimmune disease, globulin remained a significantly independent predictor of mortality of all causes and CVD whether it was evaluated by the median value or as a continuous variable (Table 2).

### Correlation between globulin and clinical parameters

The direction and strength of associations between globulin and various clinical parameters were shown in Table 3. Variables that had negative correlations with globulin included creatinine and nPNA while the levels of WBC counts, intact PTH, triglyceride and ALP showed positive associations with globulin.

**Table 3:** Multiple linear regression coefficients and Pearson correlation of globulin with various clinical parameters.

	Coefficients (95% CI)	Pearson correlation	*P* value
BMI	− 0.011 (0.98,1)	− 0.079	0.060
Creatinine	− 0.039 (0.95,0.98)	− 0.226	< 0.001*
WBC counts	0.045 (1.03,1.07)	0.196	< 0.001*
D/P creatinine at 4 h	− 0.338 (0.49,1.03)	− 0.075	0.072
nPNA	− 0.174 (0.72,0.98)	− 0.093	0.028*
Intact PTH (per 100 unit increment)	0.018 (1.001,1.03)	0.102	0.022*
Triglyceride (per 100 unit increment)	0.076 (1.03,1.14)	0.129	0.003*
Cholesterol (per 100 unit increment)	− 0.093 (0.83,1.00)	− 0.082	0.059
ALP (per 100 unit increment)	0.06 (1.001,1.12)	0.088	0.037*

*BMI* body mass index, *WBC* white blood cells, *PTH* parathyroid hormone, *ALP* alkaline phosphate, *nPNA* normalized protein nitrogen appearance, *D/P creatinine* dialysate-to-plasma creatinine ratio.

*p < 0.05.

### Predictive value of globulin for mortality compared with ferritin and WBC counts

The AUC of ROC curve for all-cause mortality was plotted and calculated to compare the predictive capacity between globulin, ferritin and WBC counts (Figs. 3, 4, 5 and 6). The AUCs of globulin, ferritin, and WBC in multivariable models were close to each other. The predictive power of globulin for 3-year mortality might be not inferior or slightly higher than that of ferritin and WBC counts (AUC 0.649, 0.607 and 0.636 for globulin, ferritin and WBC, respectively). The AUC of globulin for the overall mortality was also slightly higher than that of ferritin and WBC counts (AUC 0.646, 0.540 and 0.618 for globulin, ferritin and WBC, respectively). In the multivariate AUC calculation for the prediction of 3-year and overall mortality, similar results were produced by adding globulin to the variables in Model 4 to produce the highest AUC.

### Discussion

In the developing and developed countries, the prevalence of PD patients is on the rise. Despite significant improvements in technology and modernity, the mortality rate of dialysis patients, with CVD as the leading cause, was still estimated at almost sevenfold higher than that of the general population^2^. Because implementation of strategic treatments aimed at traditional Framingham risk factors failed to improve the clinical prognosis in ESRD patients, this realization has focused attention on nontraditional risk factors, including anemia, microalbuminuria, inflammation, oxidative stress and deranged mineral metabolism^12^. Among those novel risk factors, inflammation has been linked with adverse impact on nutritional, metabolic and cardiovascular systems. Inflammation was also reportedly to accelerate the processes of atherosclerosis in ESRD patients, and a lot of existing evidence has addressed the issue of inflammation and tried to develop anti-inflammation treatments^13^. For the first time, we found in our study that serum globulin is a new determinant of predicting overall and CVD mortality in patients undergoing PD and the association was independent of other established risk factors.

Factors contributing to chronic inflammation in ESRD can be classified as decreased renal function, dialysis-related factors, co-morbidities and intestinal dysbiosis^14^. Persistent low-grade inflammation emerges with the gradual decline in kidney function owing to accumulation of uremic toxins, increased level of endotoxin and decreased clearance of pro-inflammatory cytokines. A negative association between residual renal function and inflammatory burden was reported among dialysis patients in numerous studies^15–18^. DM caused the majority of CKD and many CKD patients were also complicated with coronary heart disease. These comorbidities can further result in higher inflammation in CKD. Unique contributors in PD include continuous exposure to biologically incompatible PD fluids, peritonitis, tunnel tract infection and exit site infection. Furthermore, the alteration of intestinal microbiota caused by disruption of normal intestinal barrier with altered permeability and the increased absorption of toxic substances produced by proteolytic bacterial species in uremia milieu leads to high inflammation^19,20^. In addition, the adipose tissue has been recognized as an endocrine organ and can exert pleiotropic effects on inflammation through its ability of secreting numerous proinflammatory cytokines. The serum levels of leptin, a protein predominantly secreted by adipocytes and excreted by the kidney, were significantly higher in PD patients than in HD patients and ESRD without dialysis, where the constant glucose load can result in the increase in fat mass over time in PD patients^21^. The underlying causes of inflammation in PD do not work independently, but rather interact with each other to amplify the degree of inflammation through a vicious circle. Moreover, the bidirectional associations between the causes and consequences of inflammation are also observed in a positive manner. Consequently, the high prevalence of inflammation in PD leaded to high cardiovascular morbidities and mortality.

The predictive role of inflammation has been thoroughly investigated in PD patients. The surrogate markers included C-reactive protein (CRP), interleukin (IL), myeloperoxidase, and tumor necrosis factor (TNF), with CRP and IL-6 being the most studied^22^. A systemic review and meta-analysis of 109 CRP studies and 22 IL-6 studies showed elevated levels of CRP or IL-6 were significantly associated with higher overall and CVD mortality in dialysis patients^23^. The disproportionately high mortality in the presence of traditional cardiovascular burden in CKD patients has been explained by protein-energy wasting (PEW) syndrome, where inflammation also plays a key role. PEW, a progressive depletion of protein and energy, is often observed in patients with CKD, particularly ESRD, and accounts for higher mortality and worsened quality of life^24^. The coexistence of inflammation makes it distinguishable from other forms of malnutrition. Inflammation not only causes an increase in nutritional requirements but also results in loss of appetite from an imbalance between orexigenic and anorexigenic mechanisms in CKD^25,26^. In the current study, we found that the protein equivalent of nitrogen appearance (PNA), the indirect marker of protein intake, was lower in patients with higher globulin levels, supporting the notion of inhibitory effect of inflammation on appetite. The existence of significant association between higher globulin and higher mortality after adjustment of nutritional indicators, such as BMI, serum albumin, and nPNA, excludes the possibility of globulin-mortality relation caused by malnutrition.

Serum globulins, one of the major constituents of total serum proteins, are believed as a good biomarker reflecting the degree and severity of inflammation and immunity because they are synthesized and secreted by the mononuclear phagocytes and mainly composed of inflammatory cytokines and antibodies. Thus, the rise in serum globulin concentrations results from the accumulation of immunoglobulins and acute inflammatory proteins. These changes are indicated of an inflammatory state as evidenced by our findings that globulin is positively associated with WBC counts in the Pearson correlation tests. Research on globulin has attracted much less interests. Li et al. in 2015 reported high preoperative serum globulin as an unfavorable survival factor in 293 locally advanced rectal cancer patients receiving neoadjunctive chemotherapy followed by radical surgery^27^. Later, a retrospective cohort study of 186 gastric cancer patients undergoing radical surgery in China evaluated the prognostic role of albumin and globulin in cancer-specific mortality, demonstrating that a high globulin level was a significant risk factor for poor survival in univariate analysis although not included in multivariate analysis^28^. The prognostic value of pretreatment serum globulin level was also shown in patients with nasopharyngeal carcinoma with a high globulin associating with a poor progression-free survival^29^. We also found serum globulin concentration as a negative survival determinant amongst patients undergoing PD.

Systemic inflammation is estimated at a range of between 12 and 65% in PD patients, depending on the cut-off value of the selected inflammatory markers^30^. Since convincing evidence confirms the detrimental impact of inflammation on clinical outcomes of PD patients, researches have been devoted to suppress the severity of inflammation both systemically and intraperitoneally. Potential therapeutic options showing promising results in preliminary studies include preserving residual kidney function, using biocompatible PD fluids, maintaining intestinal commensalism, optimizing fluid status and avoiding catheter-related infections^31^. Serum globulin level emerged as a novel biomarker for predicting mortality amongst our PD cohort. Whether the timely variation of serum globulin levels after instituting potential therapeutic interventions could reflect the changes of mortality risk requires future large-scale, prospective clinical trials.

Several limitations of this study are worth mentioning. First, routine measurements of CRP and inflammatory cytokines were not done in such a retrospective study. Although the independent role of globulin in predicting mortality among PD patients was robust in light of sensitivity tests, it remains unclear whether this relationship remains after adding some more inflammatory markers into the adjustment. Second, PD patients treated with biocompatible dialysates had a slower rate of decline of residual renal function and a higher achieved Kt/V compared with those treated with conventional solutions^32^. It is plausible that the use of bio-incompatible PD may lead to a higher risk of inflammation and death. Due to the retrospective nature of study design and technical limitation, we cannot address this important issue. Third, single measurement of serum globulin may under- or over-estimate its relations with mortality in the long term. Statistics approaches with time-varying covariates may be more optimal to clarify the relationship between globulin and mortality. Fourth, instead of being directly measured, serum globulin is calculated by the equation: (serum total protein-serum albumin). Nonetheless, our study provided the evidence that globulin is an independent variable for mortality after adjusting clinical confounders. Our method of calculating serum globulin is inexpensive and practical in clinical medical care.

Thousands of articles had been published on inflammation and dialysis because of its pivotal role in triggering the vicious cycle of cardiovascular burden, such as atherosclerosis, malnutrition, and muscle wasting, where inflammation also is magnified by positive feedback of its consequences. The independent role of serum globulin for the prediction of mortality in our study suggested that serum globulin could be used as a sensitive potential therapeutic target for various anti-inflammatory and immunomodulatory interventions. Further research is needed to elucidate whether the biological mechanism of globulin as an independent predictor of mortality is mediated by specific immunoglobulins or inflammatory proteins.

## Materials and methods

### Participants and measurements

We conducted a retrospective longitudinal study in patients with ESRD undergoing PD at a single dialysis unit of a medical center in Taiwan with the aim to evaluate the prognostic value of serum globulin level in mortality risk. Patients were considered to be enrolled in the study if they started receiving PD treatment between 2001 and 2016. Exclusion criteria included age < 18 years (n = 12) or the time on PD < 3 months (n = 12). A total of 554 patients who matched the selection criteria were eligible for study finally. The causes of deaths were collected and recorded for the analysis of cause-specific mortality. We conducted this study as per the ethnical regulations of declaration of Helsinki with the approval and surveillance of Institutional Review Board of Changhua Christian Hospital. The informed consent document from each participant was waived for a retrospective study in Taiwan.

Patients’ covariates regarding the socio-demographic characteristics, medications use, PD related data, comorbidities and laboratory variables were collected and recorded at study entry from the hospital’s database using established electronic medical records. Smoking status was classified as never, ever or current smoker, while body mass index (BMI) was derived from body weight in kilograms divided by the square of body height in meters. Clinical medical conditions consisted of diabetes mellitus (DM), hypertension, hyperlipidemia, cancer, autoimmune disease and cardiovascular disease (CVD), which was defined as the presence of coronary artery disease, cerebrovascular disease or peripheral artery disease. PD related data included normalized protein nitrogen appearance (nPNA), adequacy of dialysis (weekly Kt/V urea), 24 h urine output, and dialysate-to-plasma creatinine ratio at 4 h (D/P (creatinine) at 4 h), and residual glomerular filtration rate, which was calculated as the average of 24 h urine clearance of urea and creatinine.

Laboratory covariates used for baseline characteristics included blood levels of albumin, globulin, creatinine, glutamic-pyruvic transaminase (GPT), white blood cell (WBC) count, alkaline phosphate (ALP), hemoglobin, ferritin, cholesterol, triglyceride, intact parathyroid hormone (PTH), calcium, and phosphate. The information on pharmacotherapy included angiotensin-converting enzyme (ACE) inhibitors, angiotensin II receptor blockers (ARB), diuretics, erythropoiesis stimulating agents (ESA), vitamin D and calcium channel blockers. The study participants were categorized by the median globulin value (2.8 g/dL) as the high globulin group (≥ 2.8 g/dL), and low globulin group (< 2.8 g/dL). Globulin is derived from the difference between total protein and albumin (serum total protein-serum albumin). All patients were followed up from the date of commencing PD until death, or the end of study on 31 July 2017. Cardiovascular disease is the leading cause of death our PD cohort. The primary clinical outcome studied was mortality from all causes and CVD mortality as the secondary outcomes.

### Statistical analysis

Frequency and percentage were used to display the distribution of categorical data while means ± standard deviation (SD) or median (interquartile range, IQR) were used for continuous data depending on whether there is normal distribution, which was determined by the Kolmogorov–Smirnov test. For the comparisons of baseline patients’ characteristics between the high and low globulin groups, Student’s test or Mann–Whitney test was used for continuous data and Chi-square test or Fisher’s exact test for categorical data. Survival plot was depicted by the Kaplan–Meier estimates and the differences in survival status between groups were compared using a log-rank test. Cox proportional hazards models were conducted for multivariate adjustments to evaluate the significance between the clinical outcomes and covariates and the results were shown as hazard ratio (HR) with 95% confidence interval (CI).

We performed a hierarchical regression technique in Cox proportional hazards models to determine whether the significance between globulin and clinical outcomes was independent. The unadjusted model was constructed to determine the association between globulin and mortality risk in the univariate Cox regression model. In hierarchical framework, we built five adjustments models as follows: model 1, globulin plus age, sex, BMI, smoking status, the cause of CKD, the status ahead of PD, and educational level; model 2, variables in model 1 plus medications use; model 3, variables in model 2 plus comorbidities; model 4, variables in model 3 plus PD related data; model 5, variables in model 4 plus laboratory parameters. In this study, variables selection was non-parsimonious manner and all variables in Table 1 were considered.

The normal range of globulin was not available and the appropriate cut-off value for globulin was also undetermined. Thus, we performed three sensitivity analyses to corroborate our results. First, we re-ran the Cox analysis by treating globulin as a continuous variable. Second, the study cohort was re-grouped to three tertiles based on the globulin levels. Third, the optimal cut-off value for globulin was determined by the Receiver Operating Characteristics (ROC) curve analysis and used in the Cox models. Patients with malignancy and autoimmune disease may have increased levels of serum globulin, thus confounding our results. We repeated our analyses after excluding those with cancers or autoimmune diseases to test the robustness of our results.

The significance and strength of association between globulin and laboratory and PD related parameters were assessed by Pearson rank correlation test and multiple linear regression analyses. We calculated the area under the curve (AUC) by the ROC curve analysis to compare the mortality prediction capacity of globulin with that of ferritin, and WBC counts, both of which indicated the degree of inflammation. In addition to the comparison of AUC for individual variable, we also conducted multivariate AUC after adding the selected variable (globulin, ferritin and WBC counts) into the variables in model 4. All the statistical analyses were performed using IBM SPSS Statistics for Windows, Version 22.0 (IBM Corp., Armonk, NY). A two-sided *p* value of < 0.05 was considered statistically significant.

## Supplementary Information

Supplementary Information.

## Data Availability

The data sets generated or analyzed during the current study are available from the corresponding author upon reasonable request.
