# Supplementary material for: Serum globulin is a novel predictor of mortality in patients undergoing peritoneal dialysis
Source: Sci Rep. 2023 Jan 20;13:1139. doi: 10.1038/s41598-023-27688-z (PMC9859810; doi:10.1038/s41598-023-27688-z)
Supplement: Supplementary file 1 — Supplementary Information. [file 41598_2023_27688_MOESM1_ESM.docx]

| Supplementary table 1. The hazard ratios of mortality risk for all the variables | | | | |
| --- | --- | --- | --- | --- |
|  | All-cause mortality | | CVD mortality | |
|  | Hazard ratio (95% CI) | p-value | Hazard ratio (95% CI) | p-value |
| Globulin≥2.8 vs. Globulin<2.8 | 2.06(1.39,3.05) | <0.001 | 1.94(1.18,3.16) | 0.009 |
| Gender, male | 5.11(2.87,9.08) | <0.001 | 5.47(2.61,11.49) | <0.001 |
| Age | 1.05(1.03,1.08) | <0.001 | 1.06(1.03,1.09) | <0.001 |
| Body mass index | 1.00(0.94,1.05) | 0.923 | 1.00 (0.94,1.08) | 0.902 |
| Smoker | 0.93(0.70,1.23) | 0.602 | 0.80(0.56,1.15) | 0.225 |
| Educational level | 0.83(0.70,0.99) | 0.035 | 0.89(0.72,1.10) | 0.279 |
| Status ahead of PD | 0.84(0.52,1.33) | 0.454 | 0.62(0.34,1.15) | 0.131 |
| The causes of CKD | 0.89(0.75,1.06) | 0.207 | 0.98(0.77,1.24) | 0.862 |
| ACE inhibitor/ARB | 0.76(0.49,1.19) | 0.235 | 0.71(0.41,1.25) | 0.238 |
| Diuretics | 1.25(0.79,1.99) | 0.336 | 1.14(0.63,2.05) | 0.667 |
| Erythropoiesis-stimulating agents | 1.65(0.86,3.18) | 0.134 | 1.07(0.50,2.30) | 0.865 |
| Vitamin D | 0.89(0.53,1.49) | 0.652 | 0.55(0.27,1.15) | 0.111 |
| Calcium channel blocker | 0.78(0.51,1.2) | 0.256 | 0.62(0.36,1.07) | 0.087 |
| Hypertension | 0.58(0.34,1.01) | 0.053 | 0.62(0.30,1.26) | 0.185 |
| Diabetes mellitus | 1.61(1.03,2.53) | 0.037 | 2.18(1.22,3.90) | 0.008 |
| Cardiovascular disease | 1.05(0.69,1.59) | 0.823 | 1.18(0.70,1.99) | 0.540 |
| Hyperlipidemia | 1.07(0.69,1.67) | 0.754 | 1.19(0.70,2.02) | 0.529 |
| Cancer | 2.28(0.92,5.67) | 0.076 | 0.94(0.12,7.22) | 0.955 |
| Autoimmune disease | 1.08(0.45,2.6) | 0.863 | 1.86(0.64,5.42) | 0.253 |
| D/P creatinine at 4 hours | 0.26(0.05,1.36) | 0.111 | 0.2(0.03,1.66) | 0.138 |
| 24-hour urine volume (L) | 0.6(0.34,1.06) | 0.080 | 0.72(0.36,1.43) | 0.341 |
| Weekly total Kt/V urea | 1.26(0.72,2.2) | 0.417 | 0.96(0.48,1.92) | 0.917 |
| Daily nPNA (g/kg) | 0.61(0.35,1.06) | 0.081 | 0.62(0.31,1.22) | 0.167 |
| Residual renal function (mL/min/1.73 m^2^) | 0.85(0.69,1.05) | 0.140 | 0.91(0.7,1.17) | 0.455 |
| Serum albumin (g/dL) | 0.77(0.57,1.04) | 0.086 | 0.71(0.48,1.05) | 0.086 |
| ALP(U/L) | 1.00 (1.00,1.01) | 0.050 | 1.00 (1.00,1.01) | 0.019 |
| Calcium (mg/dL) | 1.14(0.87,1.5) | 0.340 | 1.18(0.83,1.67) | 0.354 |
| Cholesterol (mg/dL) | 1.01(1.00,1.01) | 0.002 | 1.01(1.00,1.01) | 0.001 |
| Creatinine (mg/dL) | 0.82(0.74,0.91) | <0.001 | 0.88(0.77,1) | 0.045 |
| Ferritin (ng/mL) | 1.00(1.00, 1.00) | 0.198 | 1.00(1.00, 1.00) | 0.005 |
| GPT (U/L) | 1.00(0.99,1.01) | 0.629 | 0.99(0.98,1.01) | 0.425 |
| Hemoglobin (g/dL) | 0.82(0.7,0.97) | 0.018 | 0.82(0.67,1.02) | 0.071 |
| Intact PTH (pg/mL) | 1.00(1.00,1.00) | 0.188 | 1.00(1.00,1.00) | 0.575 |
| Phosphorus (mg/dL) | 1.02(0.83,1.25) | 0.859 | 0.99(0.76,1.28) | 0.921 |
| Triglyceride (mg/dL) | 1.00 (1,1) | 0.364 | 1.00 (1,1) | 0.906 |
| WBC count (x 10^3/μL) | 1.1(1.02,1.18) | 0.008 | 1.13(1.03,1.23) | 0.006 |
| Abbreviations: ACE inhibitor, angiotensin-converting enzyme inhibitor; ARB, angiotensin II receptor blocker; BMI, body mass index; GPT, glutamic-pyruvic transaminase; WBC, white blood cell count; PTH, parathyroid hormone; ALP, alkaline phosphate; nPNA, normalized protein nitrogen appearance; D/P creatinine, dialysate-to-plasma creatinine ratio | | | | |
